# Exploring the panel and country wise effect of circular economy on environmental sustainability across six leading circular economies

**DOI:** 10.1038/s41598-026-58786-3

**Published:** 2026-06-18

**Authors:** Na Wei, Muhammad Imran, Hui Zhang, Abad Alzuman, Xuebing Zhang, Xiao Niu, Misbah Aslam

**Affiliations:** 1 School of Business, Shandong Xiehe University, Jinan, 250109 Shandong China; 2 Department of Management, College of Business Administration, Princess Nourah Bint Abdulrahman University, Riyadh, 11671 Saudi Arabia; 3 Department of Economics, International Islamic University, Islamabad, 44000 Pakistan

**Keywords:** Circular Economy, Production Based Carbon emissions, Research & Development, Panel analysis, Country wise analysis, Economics, Economics, Environmental sciences, Environmental social sciences

## Abstract

Investigating the circular economy’s impact on addressing escalating climate change concerns is crucial. The objective of this research is to examine the possible implications of a circular economy on production-based carbon emission, this domain has yet to be thoroughly investigated. This investigation fills the research gap through presenting an under examined proxy for assessing the circular economy through municipal waste generation and treatment. This research investigated data spanning 1991 to 2020 from six advanced circular economies namely, Austria, Belgium, South Korea, Spain, Sweden, and USA, applying numerous estimation techniques to ensure robust and reliable results namely Autoregressive Distributed Lag-Pooled Mean Group (ARDL-PMG), Autoregressive Distributed Lag-Mean Group (ARDL-MG), Autoregressive Distributed Lag-Dynamic Fixed Effects (ARDL-DFE), Fully Modified Ordinary Least Squares (FMOLS), Autoregressive Distributed Lag-Error Correction Model (ARDL-ECM), stability analysis and Pairwise Dumitrescu-Hurlin (D-H) causality. The study determined the influence of the circular economy on production-based emissions using both country-specific and panel data approaches. The findings demonstrate that implementing a circular economy leads to a long-term reduction in production-based carbon emissions in the panel and country wise estimation except Spain. These findings develop understanding of sustainable development difficulties and encourage practitioners to build successful strategies.

## Introduction

In the past few years, worldwide ecological issues such as biological contaminants, air pollution, and carbon dioxide (CO_2_) have become heated subjects^1^. It is difficult without eliminating CO_2_ emissions to address global warming, which significantly contribute to global climate change^2^. CO_2_ is the main factor contributing to pollution in the atmosphere (World Meteorological Organization,^3^. The seventeen Sustainable Development Goals (SDGs) designed to tackle diverse economic and environmental challenges worldwide. SDG-13 has gained attention to mitigate the detrimental effects of climate change through cutting CO_2_ emissions^4^. Therefore, this study analysis attempts to assess the impact of circular economy practices on the reduction of carbon emissions in industrial production processes. The investigation seeks to evaluate the effectiveness of circular economy concepts in reducing production-related carbon emissions across many sectors while identifying essential elements and mechanisms that facilitate this reduction.

Sustainable practices have received prodigious attention in the twenty-first century from corporations, governments, academia, and policymakers^5^. Attaining sustainable economic growth pursuant to the linear framework encounters significant obstacles owing to the Earth’s finite resources and its compromised capacity to absorb trash and pollutants. Aiming to sustain continuous economic expansion without considering the planet’s resource limitations and its capacity to handle the resultant effects poses an enormous obstacle to our goal for enduring global prosperity^6^. With the rapid growth in industrialization, natural resource consumption, and economic growth, the pressure on the global ecosystem is constantly growing ^7^, and some boundaries exceed emission and pollution levels. In this regard, the concept of circular economy (CE) is an unconventional way to boost sustainable economic growth and efficient consumption of waste and resources without compromising the green environment. The CE concept is an alternate strategy meant to lower emissions and follow to the global commitment to reduce greenhouse gas emissions. The CE model emphasizes creative techniques that challenge established conventional economic structures by reconsidering conventional waste management procedures and tackling the increasing problem of resource scarcity. Sustainable initiatives are the main demands of international policies and agreements in this regard to reducing emissions^8,9^.

Despite the lack of common consensus among different researchers on the CE concept, it is still a feasible way to manage resources. In this regard, different authors argue that it is an economic feasibility systematic shift based on the reinsertion (through biological and technological means) of used resources through material cycles^10^. It is also being described as a paradigm shift regarding resources and material use and disposal, which opposes the normal production and consumption behavior and activities of a society. Therefore, the CE is based on an economic model for human well-being and a sustainable environment^11^. This model comprises reprocessing operations, planning and designing, resourcing, production, and repurchasing to minimize waste by reproduction and boost environmental conservation^12,13^. CE is a competitive strategy for high economic development through cost-effective management, sustainable and clean technology innovation, energy efficiency, and environmental conservation^14,6,15^. Intergenerational and intergovernmental issues such as global warming, resource scarcity, and disposal of natural areas rise due to large scales of resource extraction and disposal of materials. Indeed, recycling holds a certain place in the Paris Climate Agreement, aiming to demystify climate change via a reduction of temperature to 1.5 °C and cut emissions to zero by 2050 (United Nations,^16^, therefore, in the twenty-first century, CE is rapidly gaining traction as a promising approach to address contemporary ecological challenges and mitigate their impacts on the environment^17,14^. Thus, in a nutshell, prior studies investigated the link between CE and CO^2^ emissions^18,13^, CE and GHGs^13^, CE and GHGs,^17^, and CE and sustainable development^19,20^ with indecisive outcomes. Along with CE, spending on research and development (R&D) can be seen as a driver of technological innovation, energy efficiency, and sustainable economic growth. R&D and government spending on human development and human activation upsurges human wellbeing, while the effect of R&D spending on the energy sector increases energy efficiency and mitigates CO^2^ emissions^21^. R&D in today’s technological world brings innovation in technologies, i.e., green and energy-saving technologies, which reduce energy use and emissions by enhancing efficient economic growth and consumption. Advances in science and technology increase economic development and increase information and communication technology (ICT) applications and development. ICT has shown a profound impact on human social activities, global economic development^22–24^, efficiency in productivity in industries, and human well-being and health facilities^25^. ICT either increases or decreases energy consumption and emissions, which is also unclear for diverse regions^26^.

Extensive empirical work exists on CE and diverse indicators relationships, for instance: CE and economic performance^27^, CE and sustainable development^28^, CE and emissions^13^, CE and carbon intensity, CE and eco-innovations, and CE and sustainable development goals^29^ for European Union countries. However, the findings of these studies are inconclusive and mixed. Correspondingly, other studies^30–33^ concentrated on CE and environmental well-being in different countries. Whereas other literature focused on R&D and environment^34^ for different regions with diverse findings.

This study extends the existing literature by examining the impact of the circular economy on production-related CO₂ emissions and makes several important contributions: first, it represents the inaugural attempt to quantitatively examine the relationship between the circular economy and production-based carbon emissions, at both the panel and individual country levels, thereby establishing a foundation for future research and practical applications in sustainable development. Second, this study is the inaugural effort to offer an underexamined proxy for measuring the circular economy via municipal waste generation and treatment. Third, this study adopts a comprehensive set of econometric approaches for reliable and robust results. Along with long-term estimation techniques and country-specific analysis, the additional objective is to provide enough empirical evidence and information for the establishment of the establishment of useful policies. Fourth, to the best of the authors’ beliefs, this is a first attempt to examine the statistical relationship between the circular economy, research and development expenditure, natural resources, ICT, human development, and production-based carbon emissions for the six advanced circular economies. Fifth, this study not only enhances the existing literature but also provides empirical insights, differentiating it as innovative compared to prior research endeavors. The prospective of the circular economy as a sustainable approach for alleviating climate change concerns will be evaluated. The paper will offer critical thoughts and suggestions for policymakers, industry leaders, and stakeholders about the practical application of circular economy concepts for achieving a sustainable, low-carbon future.

## Literature review

In the twenty-first century, global ecological problems have increased due to the sharp rise in industrialization, production activities, and high resource extraction and consumption activities globally^35^. To tackle the global ecological burden and comply with the Paris Agreement, a systematic shift based on the reintroducing used resources into material cycles^10^ is demanded for future generations. According to the Circularity Gap Report (2021), shifts or transitions to a CE could mitigate emissions by 39% and reduce material consumption by 28%^18^. The prior literature also shed light on CE with diverse indicators and measures as a solution to a sustainable environment. However, little attention has been given to important indicators like R&D, natural resources, ICT, human development, and transport-based CO^2^ emissions for the six advanced circular economies, and the results of the prior literature are different and unsettled in this regard.

In this regard, prior literature used diverse indicators in their studies, such as^18^ which used annual panel data and investigated the connectivity between the CE, CO^2^ emission reduction, and environmental sustainability for 29 European nations from 2000 to 2020. Using a suitable econometric method covering Heteroskedasticity and endogeneity issues, the finding discovered a positive association between CE and environmental sustainability through reducing CO^2^ emissions. The findings demonstrate that CE and recycling strategies promote sustainable development through emissions reduction.

In the context of the top-seven emission countries,^36^ found that municipal waste was positively linked with CO^2^ and desired a CE. Globalization and GDP harness CO^2^ emissions, increasing CO^2^ emissions in the top CO^2^ emission countries, while renewable energy mitigates CO^2^ emissions, increase energy security and environmental quality^37^; Özbay,^38^. Pao and Chen,^39^ probed the CO^2^ emission and CE relationship for EU countries, and findings revealed that a 1% rise in recycling reduces emissions by 0.5% in the long run. Tiwari et al.^13^ investigated the association between CE, supply chain pressure, environmental policy stringency, energy transition, and CO^2^ emissions in emerging economies. According to their study, climate policy stringency and CE enhance environmental quality by reducing CO^2^ emissions. However, supply chain pressure, industrialization, and energy transitions boost CO^2^ in both the long and short run. Razzaq et al.^40^ explore the nexus amidst GDP, MSW recycling, and CO^2^ emissions from 1990 to 2017. The findings of the study show that increased recycling constructively boosts environmental quality by reducing emissions by 0.209% (0.087%) and increasing efficient economic growth by 0.317% (0.157%). Empirical evidence discovers that cities with high CE agglomeration achieve significant reductions in per capita CO^2^ emissions, demonstrating CE’s effectiveness in fostering low-carbon growth. Di,^41^ argued that the growth of the CE is necessary for the reduction of carbon emissions with urban development, industrial rationalization, and the industrial resource cycle acting as key mediators. The findings emphasize the importance of tailored strategies and inter-regional cooperation to achieve sustainable carbon reduction.

Gallego-Schmid et al.^17^ probed CE and climate change for a sustainable environment. The main focus of this research was on slowing resource solutions to diminish emissions by 99% per functional unit, and in this regard, CE, or reuse of material, is the most appropriate alternative. The closing reuse approach can mitigate GHG emissions by 30–50% per functional unit, while the remaining are based on recycling efficiency. Schwarz,^42^ findings for 15 polymers from European Union (EU) countries indicate that recycling mitigates CO^2^ emissions by 73%. Another 25 EU-based studies from 2010 to 2019,^19^ investigated the link between CE sources of value (recycling, repair, reuse, and renewable energy) and sustainable development, and the outcomes revealed that CE positively impacts a sustainable environment; however, the impacts of CE sources of value show diverse findings. On the other hand, Bayar et al., (2021) ^92^ explore renewable energy, municipal waste recycling, and environmental sustainability for EU countries from 2004 to 2017 by using causality analysis. The outcomes determine an insignificant relationship between recycling rate, renewable energy, and CO^2^ emissions. Similarly,^43^ highlight the importance of economic and environmental assessments, supply risks, and the potential of CE strategies in addressing the challenges associated with critical metals in EV batteries and carbon emissions mitigation. Several analyses revealed that EV batteries invented by China can be reused and recycled, along with a supportive guard against carbon emissions.

Using the dataset from 1998 to 2021,^44^ linked R&D, natural resources rent, ICT, and CO^2^ emissions under the sustainable development goals. The innovative findings of the study show natural resources, economic growth, and urbanization upsurge CO^2^ emissions, whereas R&D, ICT, and clean energy sources mitigate CO^2^ emissions and support a sustainable theme. An inverted link was found between natural resources and CO^2^ emissions. In addition, the findings of the study are robust through diverse estimation techniques such as panel Panel regression, augmented mean group, and common correlated effect mean group. In a similar work,^45^ explored the association between R&D, technology innovation, and CO^2^ emissions in G-7 countries for the years 1990–2020. Technological advancement diminishes CO^2^ emissions. Similarly, R&D and renewable energy consumption diminish CO^2^ emissions. Imran,^44^ explored the relationship between R&D, energy efficiency, ICT, and environmental sustainability in Sweden by using the Quantile-on-Quantile Kernel-Based Regularized Least Squares (QQKRLS) analysis. The study underlines the constructive relationship between R&D, energy efficiency, ICT, structure change, CO^2^ emissions reduction, and enhancing environmental quality. Özbay,^46^ analyzed data from around 16 OECD nations spanning from 1990 to 2019. The study utilized fully modified least squares, mean group dynamic least squares, and FGLS estimation methods. The results indicated that research and development investment pertaining to the environment enhances environmental wellbeing.

Industrial restructuring, R&D, technology innovation, and the CO^2^ emissions link are examined by^47^ in 30 Chinese provinces from 1999 to 2020. The results demonstrate that technological advancement boosts carbon emissions efficiency. While the moderating effect of R&D reduces emissions in India,^48^ analyzed R&D, green patents, energy, and CO^2^ emissions in the China Pilot Low Carbon City Program (CPLCCP) by adopting the CS-ARDL, Q-ARDL, and Ganger’s non-causality approach for 118 Chinese cities over the time frame of 2003–2020. The estimated outcomes portray the positive association of R&D and technological advancement with environmental sustainability. The findings support the presence of the environmental Kuznets Curve (EKC) hypothesis. You et al.^49^ examined the link between technology innovation, renewable energy, human capital, and CO^2^ emissions for 64 BRI countries from 2000 to 2021. The augmented mean group (AMG) estimator and mean group (MG) findings show an inverse relationship between ICT, REC, human capital, and CO^2^ emissions in BRI regions. However, the interaction term of EG with human capital, REC, and ICT mitigates CO^2^ emissions, and the U-shaped EKC is found among ICT and CO^2^ emissions. In another study by applying the NARDL technique,^50^ probed natural resources, ICT, and renewable energy consumption for sustainable development in China from 1990 to 2021. The interesting outcomes of the study show that a negative shock to natural resources increases CO^2^ emissions and vice versa. FDI and ICT boost CO^2^ emissions in China. These links are confirmed through an additional causality analysis of the study. Özbay and Duyar,^51^ gathered data from 20 OECD nations and utilized Fully Modified Ordinary Least Squares and Mean Group Dynamic Least Squares methodologies. The results revealed that higher education has a negative correlation with carbon emissions.

This study is distinct in relation to proxies and the dependent variable. For instance,^18^ utilized the recycling rate to assess its effect on carbon emissions. Tiwari et al.^13^ analyzed municipal waste generation to evaluate its influence on carbon emissions. Pao and Chen,^39^ included many variables to assess the circular economy, except the proxy utilized in our analysis. This research uses municipal waste generation and treatment to assess the circular economy while also including production-based carbon emissions instead of only focusing on carbon emissions. This study examines both panel-based and country-specific analyses to provide a comprehensive view on the circular economy and production-based carbon emissions, in contrast to the discussed studies that concentrated solely on panel-based research.

### Theoretical framework

Production-based carbon emissions (PBC) signify the emissions caused across national or sectoral borders during the manufacturing of products and services. As a dependent variable, PBC functions as a vital metric for climate policy, encompassing the ecological expenses associated with domestic production. The circular economy proposes an alternative framework that prioritizes resource conservation, waste reduction, and closed-loop systems^52^. The CE approach supposedly curbs PBC by encouraging concepts like reduction, reuse, recycling, and recovery, which lessen raw material consumption, prolong product lifecycles, and improve the utilization of secondary resources^53^. This transformation eliminates industry dependence on environmentally damaging production, therefore reducing emissions at the source. In accordance with ecological modernization theory (EMT), technological advances and sustainable industrial practices within a CE framework have the potential to separate economic progress from environmental harm^54^. Similarly, The Porter Hypothesis argues that stringent environmental policies, including circular resource utilization, might encourage invention and enhance the economy while decreasing PBC^55^. The conceptual expectation posits that increased adoption of circular economy approaches would result in a major reduction in PBC^56^. This approach posits those circular approaches function through both technological tools and administrative changes, resulting in systemic modifications within production networks. The study asserts an adverse association between the implementation of a circular economy and production-related carbon emissions. The notion of “Dematerialization” stimulates creativity via ICT. This notion underlines the utilization of digital resources, automated workflows, and the minimization of dependence on physical resources to reduce carbon emissions^57^. Resource rents derived from natural resources are a substantial income source. Inadequate management of natural resources can result in economic disparities and environmental degradation, whereas the equitable distribution of profits can serve as a financial resource for effective ecological management^58,59^. Investment in research and development enhances environmental advancement by encouraging policies that include human capital development and sustainability^60^.

## Methodology

**Fig. 1:**
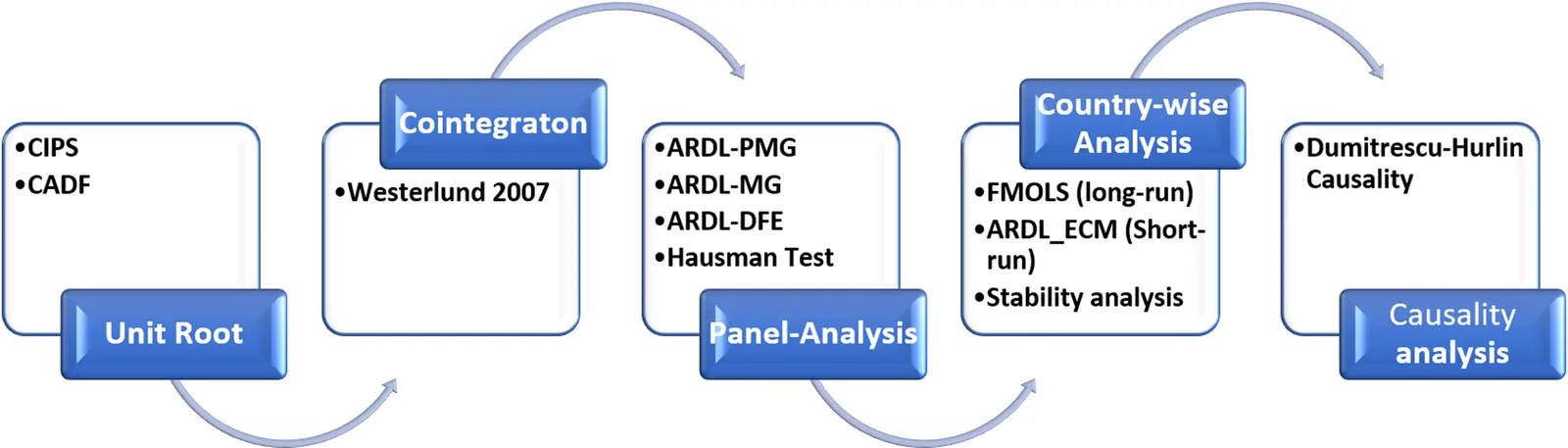
The research methodological flow.

Initial empirical tests, including unit root and cointegration tests, were carried out to ensure the appropriateness of the data for further analysis. Upon confirming the diagnostic tests, the study proceeded with panel data analysis techniques. Specifically, the ARDL approach was employed, with variations such as PMG, MG, and DFE to account for different aspects of the data and provide a comprehensive understanding of the relationships between variables^61^. The structure and flow of the research can be visually represented in Fig. 1.

### Data

**Fig. 2:**
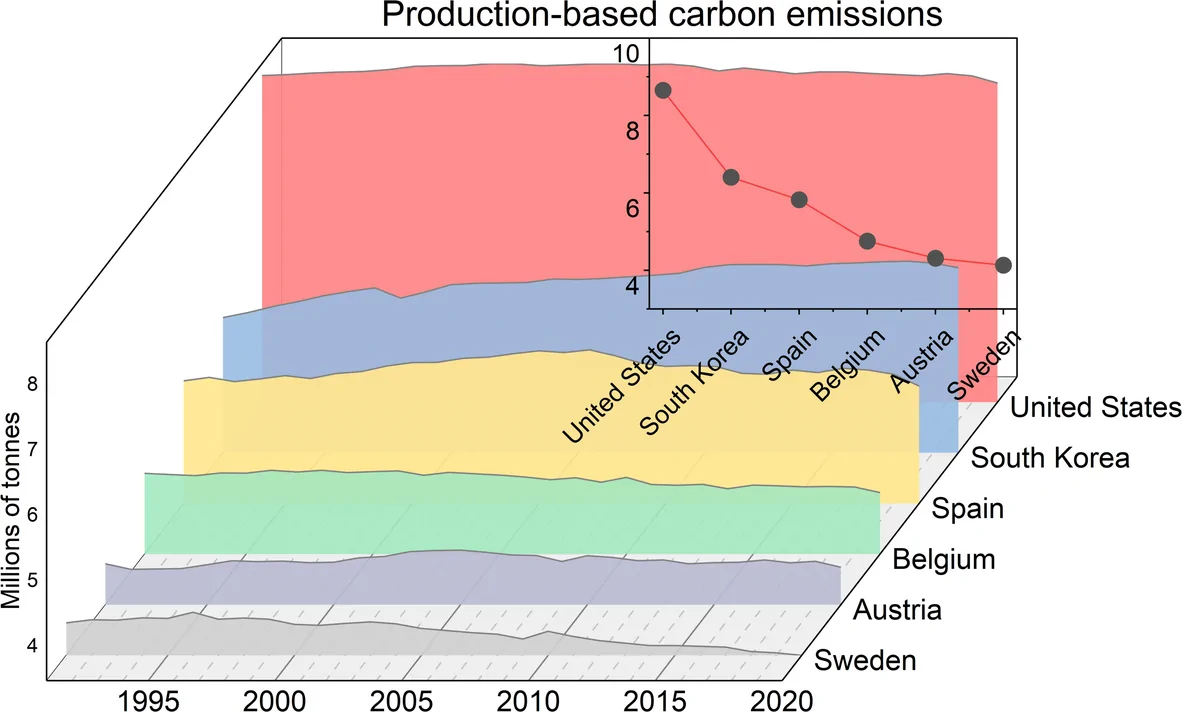
Production-based carbon emission trends from 1991 to 2020.

**Fig. 3:**
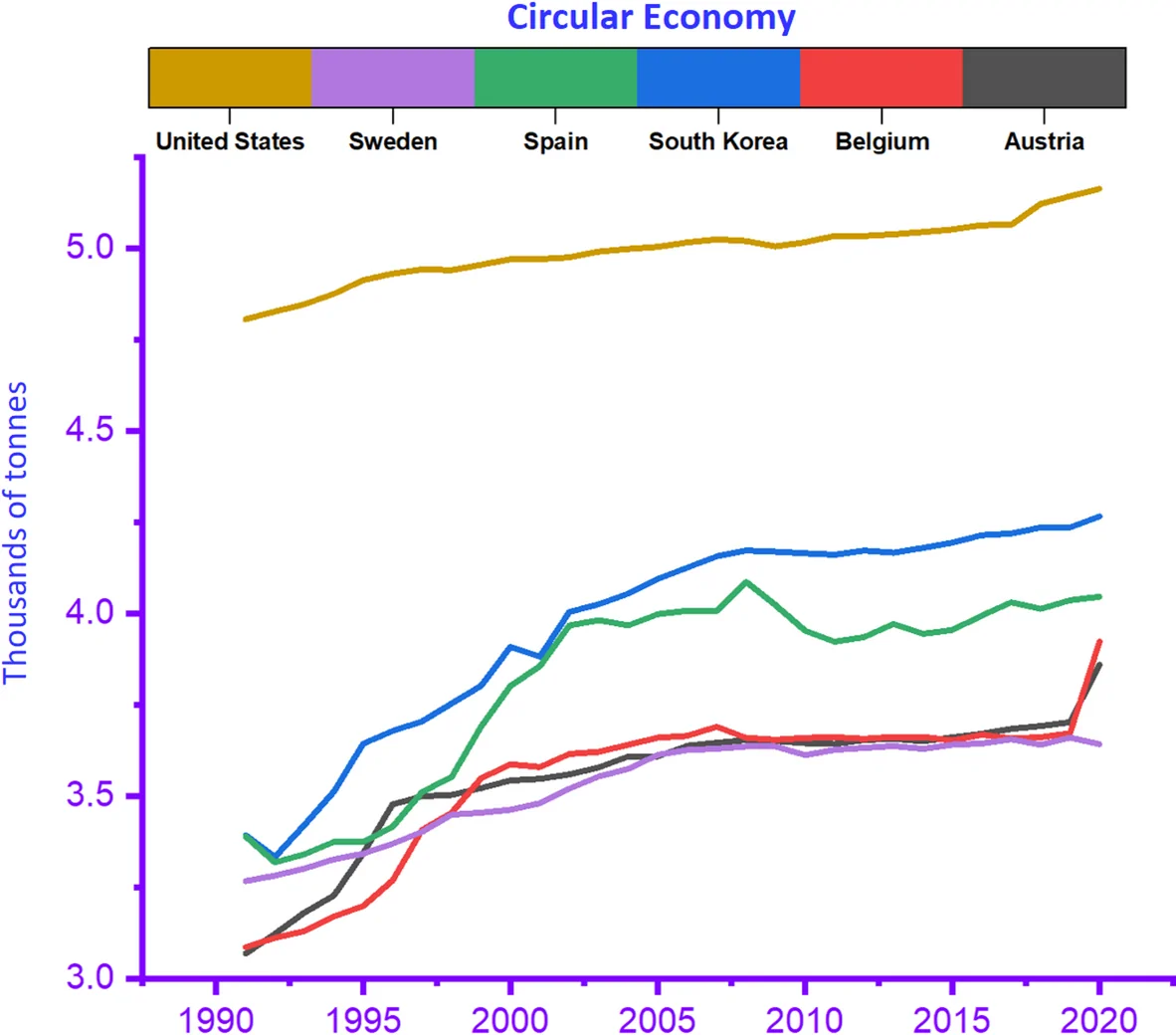
Evolutionary trends in circular economy from 1991 to 2020.

**Fig. 4:**
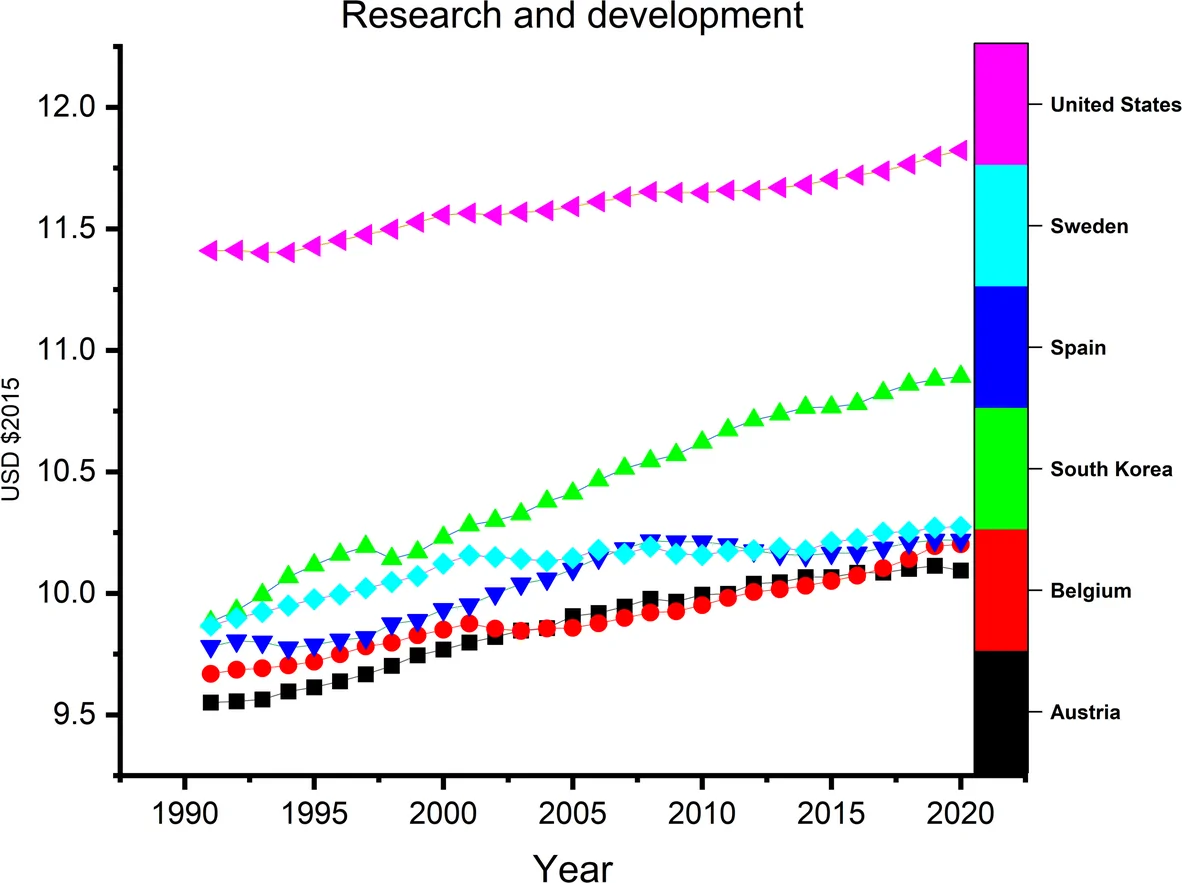
Illustrates the trends of research and development expenditure from 1991 to 2020.

**Fig. 5:**
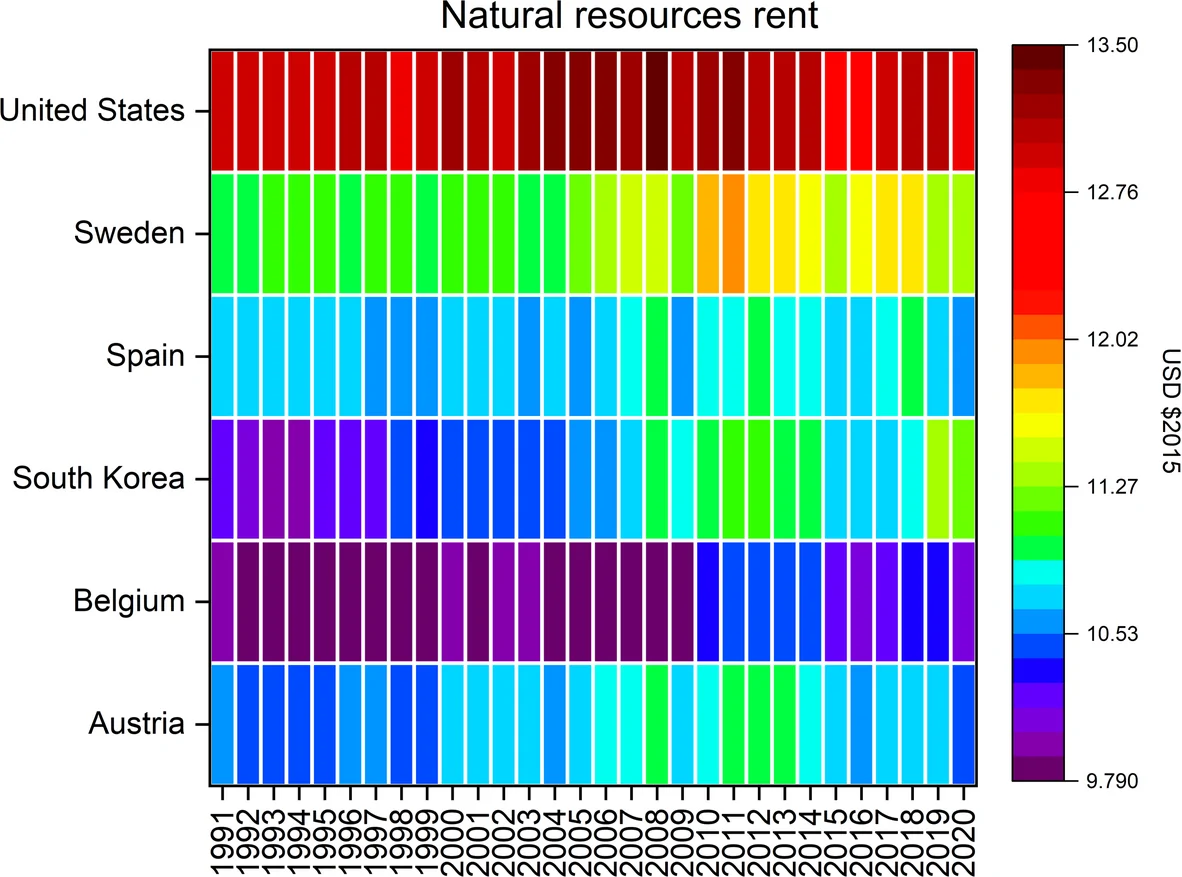
Temporal analysis of natural resources rent from 1991 to 2020.

**Fig. 6:**
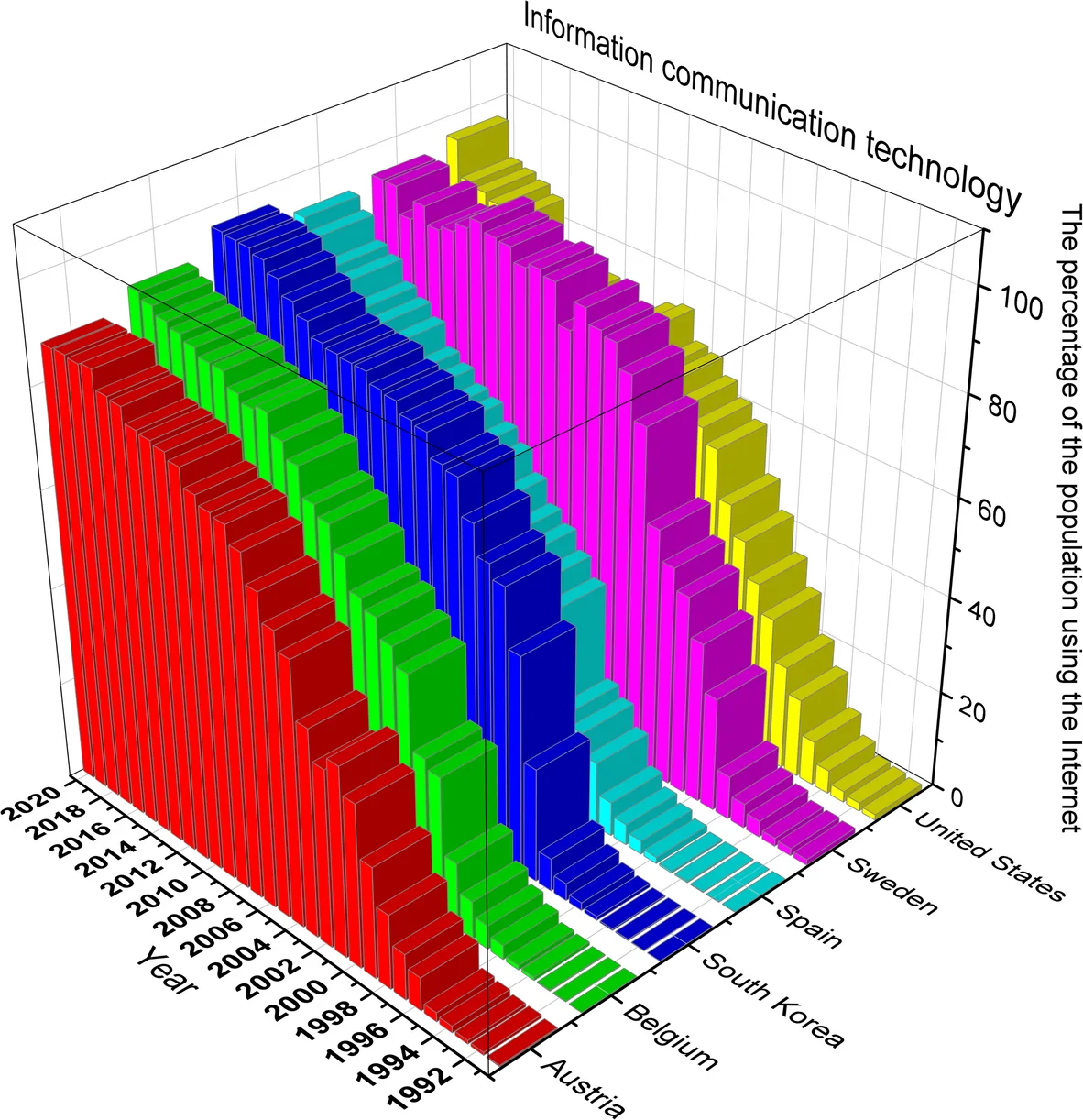
Showcases the progressive development ICT throughout the years 1991 to 2020.

**Fig. 7:**
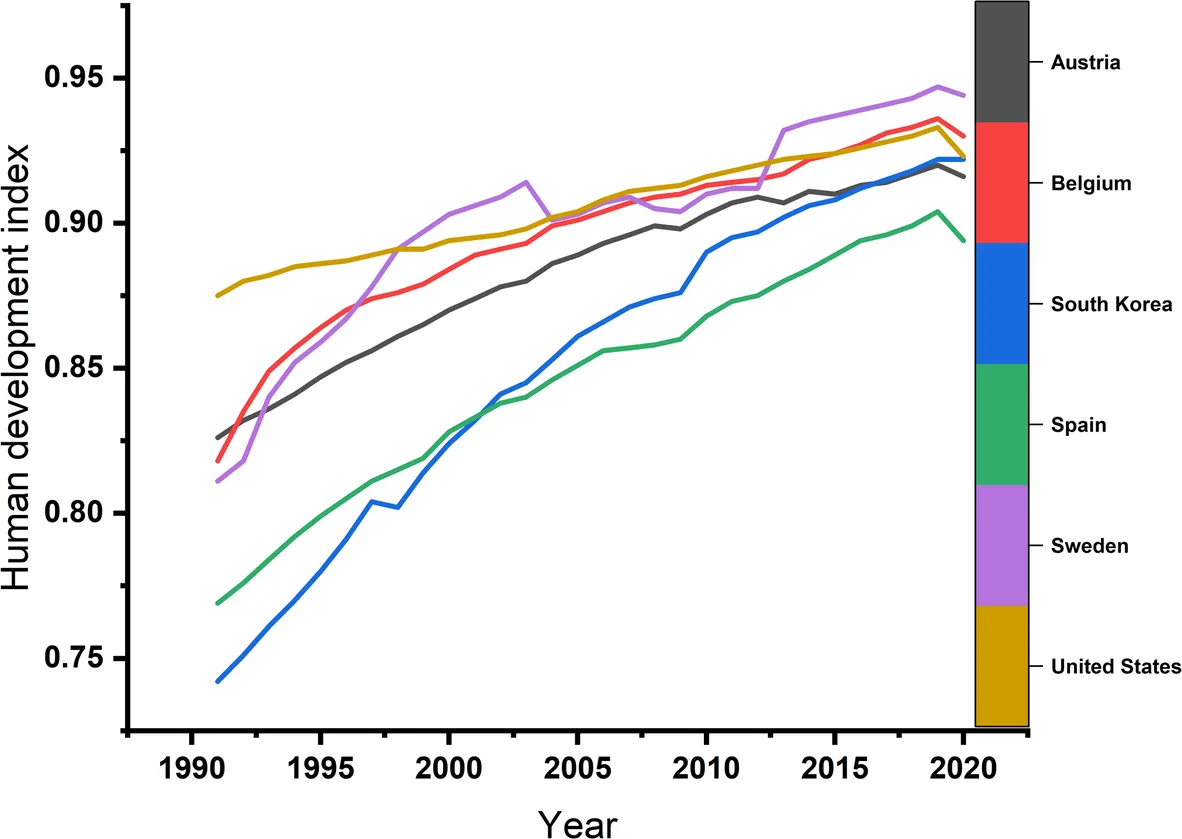
Depicts the human development from 1991 to 2020.

The data for carbon emissions, CE indicators and R&D were obtained from the Organization for Economic Co-operation and Development database (OECD,^62^; OECD,^63^. Meanwhile, ICT, and Natural resource rents were extracted from the World Development Indicators database (World Bank,^64^. Lastly, information on human development was sourced from the United Nations Development Programme’s database^65^. Table 1 reports the summary information of all variables, including their names, abbreviations, measurement units, and corresponding data sources. The study selected countries based on the maximum and most comprehensive data available regarding the proxy utilized in this research to assess municipal waste treatment from 1991 to 2020. Figures 2, 3, 4, 5, 6 and 7 presenting the visual presentation of production-based carbon emissions, circular economy, and participating variables in logarithm form. Figure 2 represents production-based carbon emissions in six economies engaged in circular economies. The USA and South Korea have evolved into high-production, pollution-intensive economies, with significant emissions relative to other prominent circular economies. Figure 3 outlines the increasing trends in the management of municipal waste creation and treatment, underlining the United States, South Korea, and Spain as the foremost countries in integrating recyclables into their economies. Figure 4 provides a regular rise in R&D development, with the USA, Sweden, and South Korea appearing as significant global leaders in this field. Figure 5 summarizes the natural resources rent among the 06 economies, revealing that the United States and Sweden reflects the greatest number of natural resources rent relative to the other studied economies. Figure 6 specifies the proportion of the population accessing the Internet, indicating that all selected studies report over 88% of their population accessing the Internet, so illustrating the expansion of access to the internet and the further development of ICT growth. Figure 7 highlights that Belgium and Sweden are among the best in the HDI index, with both nations achieving an index of approximately 0.92 out of 1 and experiencing a continuous growth trend.

This multifaceted dataset, compiled from reputable international sources, allows for a comprehensive analysis of the interconnected factors that influence carbon emissions and the potential impact of a circular economy on environmental sustainability.

**Table 1:** Variables description.

Variable	Symbol	Measuring unit	Data sources and links
Production based carbon emissions	PBC	Millions of tonnes	(OECD,^62^
Circular economy	CE	Thousands of tonnes	(OECD,^62^
Research and development expenditure	RD	Constant $ 2015	(OECD,^63^
Natural resource rents	NR	Constant $ 2015	(World Bank,^64^
Information communication and technology	ICT	Individuals using the Internet (% of population)	(World Bank,^64^
Human development	HDI	Index	United Nations Development Programme,^65^

### Variable definition and measurement

“Production-based carbon emissions” refers to the aggregate greenhouse gases (mostly CO₂) emitted within the territorial limits of a country as a result of the production of products and services. It measured in millions of tonnes. The circular economy is a production and consumption model that emphasizes the reuse, repair, transformation, and recycling of resources and goods for as long as feasible, represented by municipal waste generation and treatment, measured in thousands of tonnes, Research and development expenditures comprise both current and future funding (from public and commercial sectors) dedicated to systematic creative endeavors with the goal of enhancing competitiveness and its application for novel uses. Research and development, measured by the total volume in 2015, expressed in US dollars. Natural resource rent pertains to the excess value produced from the extraction or utilization of a natural resource. Natural resource rent, indicated by the total volume in 2015, expressed in US dollars. Information and communication technology, measured as the percentage of the population using the Internet. This is demonstrating the degree of digital connectivity and technology integration in the community. Human development index, represented by an index score. This evaluates a country’s average performance across three fundamental elements of human development: lifespan and health, educational access, and a satisfactory level of standard of living. In order to facilitate the analysis and comparison of the variables, all the units of measurement were transformed into their logarithmic form. The use of logarithmic values allows for the interpretation of the estimated coefficients as elasticities, providing a clearer understanding of the relative impacts of changes in each variable on the dependent variable.

These varying units of measurement provide a diverse and comprehensive representation of the different factors under investigation, allowing for a thorough analysis of their interconnections and impacts on the overall research objectives.

### Econometric approach

This study aims to contribute meaningfully to the ongoing discourse on carbon emission reduction by examining the intricate linkages between the circular economy and PBC, while incorporating key control variables such as research and development, natural resource rents, ICT, and human development. To accomplish this, we developed an econometric model, represented in Eq. (1), which draws on a thorough examination of recent studies in the field. By meticulously following established methodologies, we aimed to ensure the validity and reliability of our conclusions and provide actionable insights for policymaking and industry practices.

1 $$\:log{PBC}_{it}={\beta\:}_{0}+\:{\beta\:}_{1}log{CE}_{it}+\:\sum\:{\beta\:}_{n}{Controls}_{it}+{\:\epsilon\:}_{it}$$

Our efforts focus on enriching the ongoing discussion with new perspectives and empirical evidence, ultimately contributing to the pursuit of more sustainable and environmentally responsible practices in the face of complex and interconnected factors namely research and development, natural resource rent, ICT and human development. Upon incorporating the control parameters into the equation, the final model can be represented as follows:

2 $$\:log{PBC}_{it}={\beta\:}_{0}+\:{\beta\:}_{1}log{CE}_{it}+\:{\beta\:}_{2}log{RD}_{it}+\:\:{\beta\:}_{3}log{NR}_{it}+\:\:{\beta\:}_{4}log{ICT}_{it}+\:\:{\beta\:}_{5}log{HDI}_{it}+\:{\epsilon\:}_{it}$$

In this equation, the variables ‘i’ and‘t’ represent the country and the particular year, respectively. β1 signifies the estimated coefficient of the primary variable, while βn denotes the coefficients of the control variables. The error term is represented by ε.

The variable logPBC stands for production-based carbon emission, logCE signifies circular economy, logRD denotes research and development, logNR reports natural resource rents, logICT represents information communication technology and logHDI represents human development index.

### Panel long run estimation

In order to investigate the nature of the connection between the variables, this research utilized a number of different estimation methods, specifically ARDL-PMG, ARDL-MG, and ARDL-DFE. Following the implementation of these techniques, a Hausman test was carried out in order to ascertain the method that was the most suitable based on the outcomes of the test. A comparison of the estimations was made possible by the Hausman test, which also assisted in determining the method that was most suitable for the data and the objectives of the research undertaking. In order to conduct additional analysis and interpretation of the findings, the technique that was selected was initially utilized. This approach ensures the selection of the most appropriate estimation technique, taking into consideration the particular characteristics of the dataset and the research questions, which ultimately results in results that are more accurate and reliable. The panel ARDL approach gives valid and effective insights even with limited sample sizes^66^. ARDL-PMG accepts different orders of integration (I(0) or I(1) within the panel unit root framework^67^. It handles endogeneity issues through explicitly involving dynamic adjustments and lag structures in the long-run regression estimation^68^. The application of this estimation technique has been inspired by its recent utilization in the works of the these researchers (Adefeso & Muraina, 2024). Its distinctive feature supports an accurate and consistent computation of long-run coefficients, even in scenarios with few data points^69^. It acknowledges the probable variability that may exist across different panels. Their successful implementation of the method served as a motivating factor for its adoption in the current study.

### Country wise estimations

To perform a country-wise long-run analysis, the study employed the Fully Modified Ordinary Least Squares (FMOLS) method. FMOLS addresses the issue of endogeneity in long-term regression estimates^70^. The main aim of this estimation procedure is to eradicate serial correlation and endogeneity bias, hence enabling conventional inference^71^. For the country-wise short-run analysis, the Autoregressive Distributed Lag – Error Correction Model (ARDL-ECM) technique was utilized. This technique employed the error correction model (ECM) with ARDL, as recently executed by^72^, to determine the country-specific impact of the short-run elasticity of the explanatory factors on the dependent variable. To further ensure the reliability and stability of the results, the study incorporated a stability analysis. This additional step enhances the overall credibility of the findings. By combining FMOLS, ARDL-ECM, and stability analysis, the research offers a comprehensive examination of both short- and long-run effects at the country level, ensuring a thorough understanding of the relationships between the studied variables and their potential implications for policy and practice.

## Results and discussion

### The outcomes of descriptive stats

Table 2 provides an overview of the descriptive statistics for the six variables examined in this study. It reports the statistics for each variable. The mean values range from − 0.0559 (logHDI) to 12.3302 (logRD), while the median values range from − 0.0491 (logHDI) to 12.1433 (logRD). The maximum values vary from 1.9846 (logICT) to 13.8274 (logRD), and the minimum values range from − 1.6974 (logICT) to 9.7954 (logNR). The standard deviations span from 0.0216 (logHDI) to 0.9915 (logNR), highlighting differences in data dispersion across the variables. These descriptive statistics give an overview of the data distribution, central tendency, and variability for each variable. This information lays the groundwork for the further analyses and modeling approaches presented in the following sections of this research article.

**Table 2:** Descriptive statistics.

	logPBC	logCE	logRD	logNR	logICT	logHDI
Mean	2.3774	3.9010	12.3302	11.0747	1.3761	-0.0559
Median	2.1775	3.6622	12.1433	10.7586	1.8214	-0.0491
Maximum	3.7581	5.1638	13.8274	13.4996	1.9846	-0.0237
Minimum	1.5092	3.0697	11.5506	9.7954	-1.6974	-0.1267
Std. Dev.	0.6924	0.5550	0.6249	0.9915	0.8623	0.0216

### The result of unit root

The results presented in Table 3 reveal a heterogeneity in the integration order of the variables for the entire sample of six advanced circular economies. According to the 2nd generation CIPS and CADF tests developed by^73^, a mixed order of integration exists among the studied variables.

**Table 3:** The unit root test.

Variables	CIPS		CADF	
	Level I (0)	1st Diff. I (I)	Level I (0)	1st Diff. I (I)
logPBC	-2.176	-5.623*	-1.450	-4.229*
logCE	-2.098	-3.927*	-2.181	-2.746*
logRD	-1.382	-3.966*	-1.415	-2.690*
logNR	-1.991	-4.887*	-1.962	-4.477*
logICT	-3.209*	-5.113*	-3.293*	-3.755*
logHDI	-2.809*	-4.976*	-2.435*	-3.368*

Note: *, ** & *** demonstrate 1%, 5%, and 10% levels of statistical significance, respectively.

### The result of cointegration

To examine the long-term association, the study employs 2nd generation panel cointegration tests in conjunction with bootstrapping probabilities. The findings of the^74^ cointegration test, as illustrated in Table 4, affirm the long-term cointegration of all the chosen variables.

**Table 4:** Bootstrapping Westerlund ECM panel cointegration tests.

Statistics	Value	Z-Value	*P*-Value	Robust *P*-value
Gt	-3.731**	-1.907	0.028	0.030
Ga	-12.311	1.847	0.968	0.130
Pt	-9.094**	-2.279	0.011	0.020
Pa	-11.077	1.250	0.894	0.210

Note: The symbols *, ** & *** indicates 1%, 5% and 10% level of significance, respectively.

### The result of panel analysis

Table 5 highlights the outcomes of ARDL for a sample of six advanced circular economies over long-run. The findings obtained from ARDL report the obvious connectivity of decomposed logCE on the magnitude of logPBC. A detailed examination of logCE and estimated coefficient values reveals that the magnitude of logPBC changes caused by -0.1326 at 5% significance level. The implications of this study propose that a small 1% change in logCE would lead to a 0.1326% favorable change in logPBC in the longer term. Various elements inherent to six advanced circular nations play a crucial role in advancing the sustainability. These factors help solidify the underlying logic supporting the progression and expansion of sustainable development initiatives. First, as recycling becomes more widespread, industries in these countries become more resource-efficient by reusing materials and decreasing the need for energy-intensive primary production, which results in lower carbon emissions. Second, the adoption of advanced recycling technologies in these countries likely enhanced the efficiency of recycling processes and decreased energy consumption, ultimately leading to lower carbon emissions. Third, the presence of well-developed waste management systems in these countries enables efficient and large-scale recycling practices, helping to reduce production-based carbon emissions. Fourth, collaborative efforts across industries, academia, and governments foster the development of innovative recycling solutions and enhanced waste management practices, thereby reducing carbon emissions. Fifth, advances in recycling technologies and techniques in these countries have enabled the processing of waste materials with lower energy consumption, thereby reducing the overall carbon emissions associated with recycling.

**Table 5:** Panel Long-Run Estimation for 06 circular economies from 1991 to 2020.

Variables	PMG		MG		DFE	
	Coeff.	Z-Value	Coeff.	Z-Value	Coeff.	Z-Value
*Long-run*						
logCE	-0.1326**	-2.43	-0.1019	-0.70	0.4630*	4.77
logRD	-0.0992**	-2.05	-0.2776	-0.87	0.2188*	2.83
logNR	-0.0285*	-2.80	0.0316	1.10	-0.0317	-1.12
logICT	0.0893*	7.16	0.0842*	2.98	-0.0707*	-3.22
logHDI	-4.4360*	-6.97	0.7219	0.14	-2.4792**	-2.50
*Short-run*						
logCE	0.2861	1.19	0 0.2128	1.29	-0.3758*	-3.47
logRD	0.0774	0.43	0.2236	6.57	0.2474*	6.34
logNR	0.0275**	2.16	-0.0006	-0.04	0.0388	1.36
logICT	-0.0652**	-2.15	-0.0411*	-4.21	0.1072*	2.56
logHDI	1.4582	1.09	1.8215	1.37	4.5216**	2.19
ECT_t−1_	-0.5351*	-2.89	-0.8110*	-6.15	-0.7838*	28.69
Constant	2.1905*	2.94	3.1181***	1.84	-1.4294***	-1.87
Hausman Test		0.7450				1

Note: *, ** and *** illustrate statistical significance level at 1%, 5% and 10% respectively.

Our results are further validated by various reports and research literatures that support our findings. Such as recycling leads to various environmental improvements, particularly by redirecting waste from landfills and mitigating the emission of pollutants. Additionally, it aids in fulfilling the material demands of economic activities, thereby mitigating the environmental consequences related to raw material acquisition and processing^14,75^. Implementation of efficient waste management strategies and adoption of water conservation practices to minimize water use and safeguard natural resources, all of which can be achieved through recycling, contribute to promoting sustainability^76^.

In the context of using logRD as a control variable for examining its connection with logPBC, it is worth mentioning that the logRD elasticities show a decline of 0.0992 in logPBC because of logRD usage in the long-run. Research and development and innovations can lead to diminished energy use and waste creation, subsequently decreasing production-based emissions, as well as uncovering novel methods to minimize, repurpose, and recycle materials in the manufacturing process. The findings on the relationship between research and development and carbon emissions in this investigation corroborate the results observed in prior studies^77^.

A 1% increase in natural resource rents correlates with a 0.0285% decrease in production-based carbon emissions in the long-run, indicating a modest but notable impact on the environment. This statement highlights a notable but moderate connection between higher natural resource rents and decreased carbon emissions in the context of production processes. Essentially, as more financial returns are obtained from utilizing natural resources, production activities lead to slightly diminishing of greenhouse gas emissions. Investing natural resource rents in the development of recycling industries and waste management infrastructure can improve energy efficiency, leading to lower overall energy consumption and reduced carbon emissions. The findings of the study are consistent with previous research, which suggests that natural resource rents can contribute to a reduction in carbon emissions once a specific threshold is reached^9^.

The 0.0893% variance in PBC resulting from a 1% increase in ICT indicates a growth in carbon emissions. The positive effect of ICT, evidenced through internet usage, on carbon emissions may correspond to heightened energy consumption resulting from digital activities such as data transmission, cloud computing, and an increasing number of digital services. Increased internet penetration also enhances consumption, e-commerce, and the utilization of digital infrastructure, all of which lead to greater energy consumption and emissions. These findings are consistent with earlier studies that associate ICT usage with elevated carbon emissions (Batool et al., 2022)^93^.

Production-based carbon emission is highly susceptible to variations in human capital development, which can significantly worsen environmental conditions. This finding aligns with previous studies suggesting that enhancing human capital leads to increased economic activity and, consequently, greater environmental impact.

Conclusively, the research employed the Hausman test to determine the most suitable estimation approach among PMG, MG, and DFE. Initially, the study compared PMG and MG, ultimately selecting PMG due to its Hausman probability value exceeding 5%. Subsequently, the Hausman test was performed between PMG and DFE, resulting in PMG being selected as the final model based on the test’s outcome. This systematic process ensured the selection of the most appropriate model for accurate and reliable estimation. Moreover, the ECT values of all three models being less than 1 provides additional support for the robustness and dependability of the long-run interaction among the studied variables. This finding strengthens the chosen model and its capacity to accurately capture the dynamics of the variables over time, instilling confidence in the reliability of the results obtained from the analysis.

### Country-wise Long-Run Estimation for 06 circular economies from 1990 to 2020

**Fig. 8:**
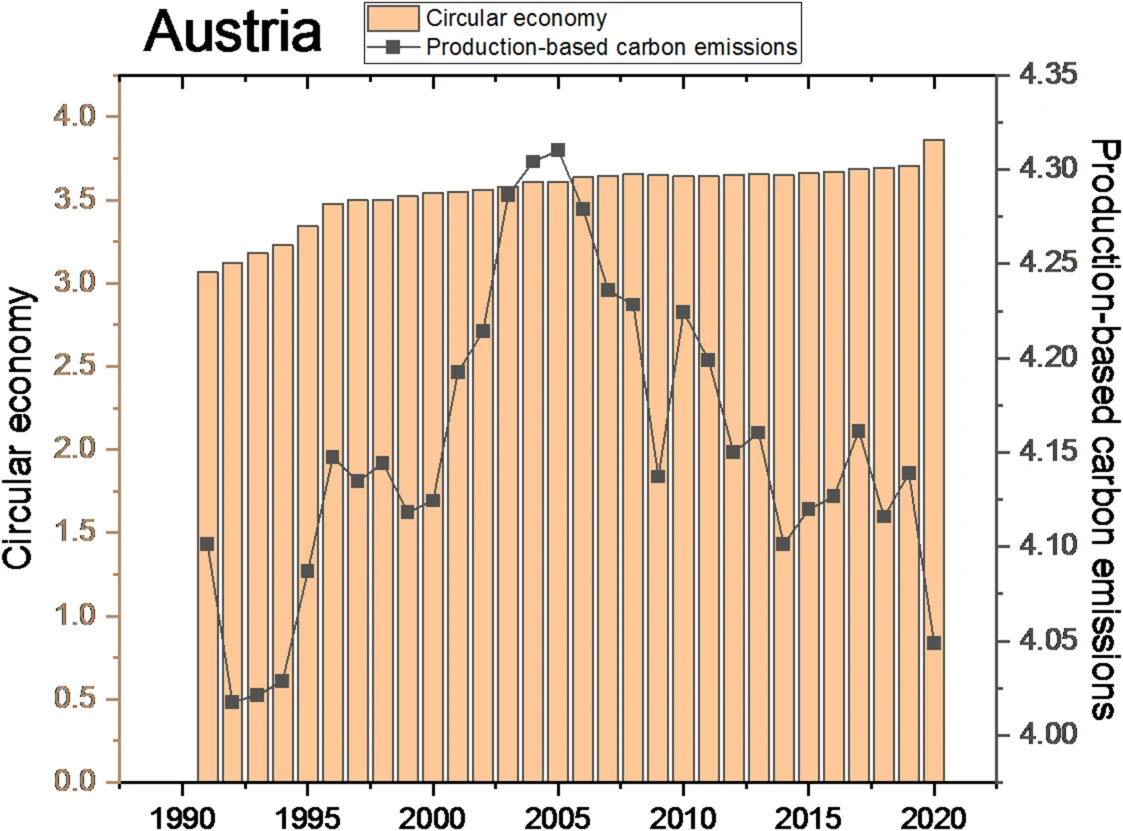
The graph illustrates PBC and CE trends of Austria from 1991 to 2020.

**Fig. 9:**
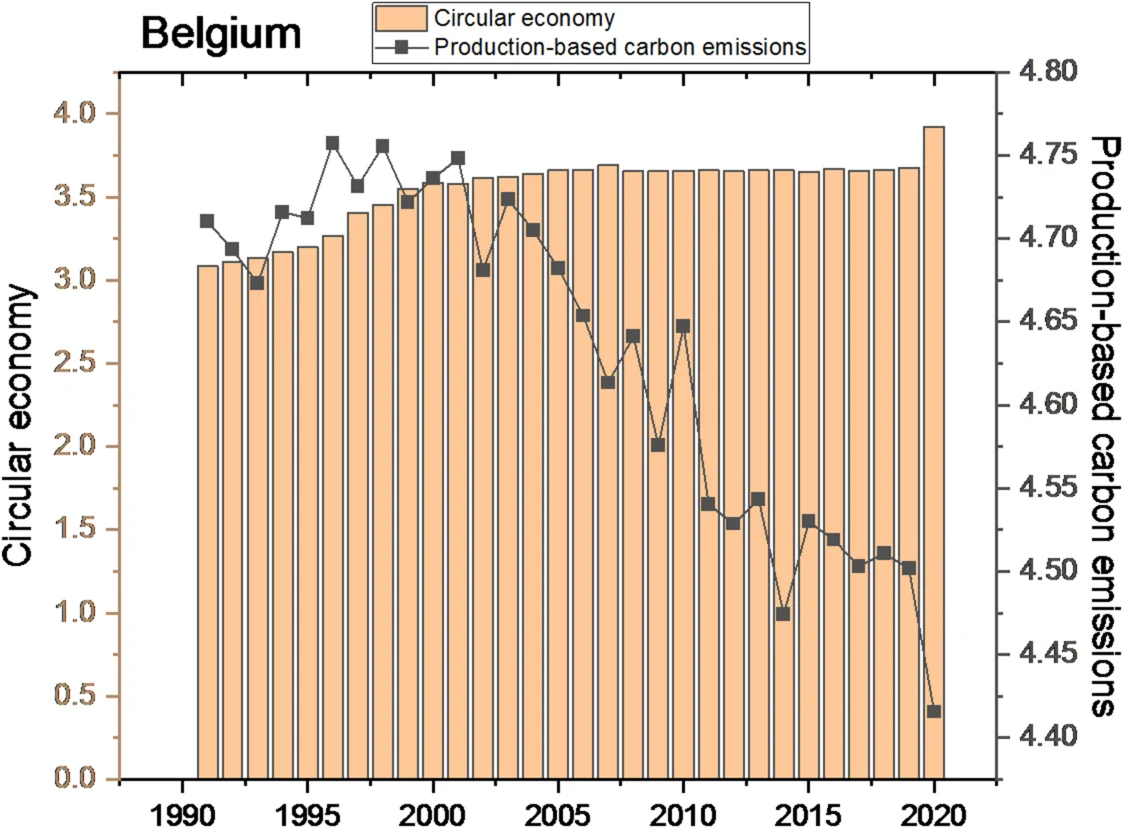
Visualizing PBC and CE correlation of Belgium from 1991 to 2020.

**Fig. 10:**
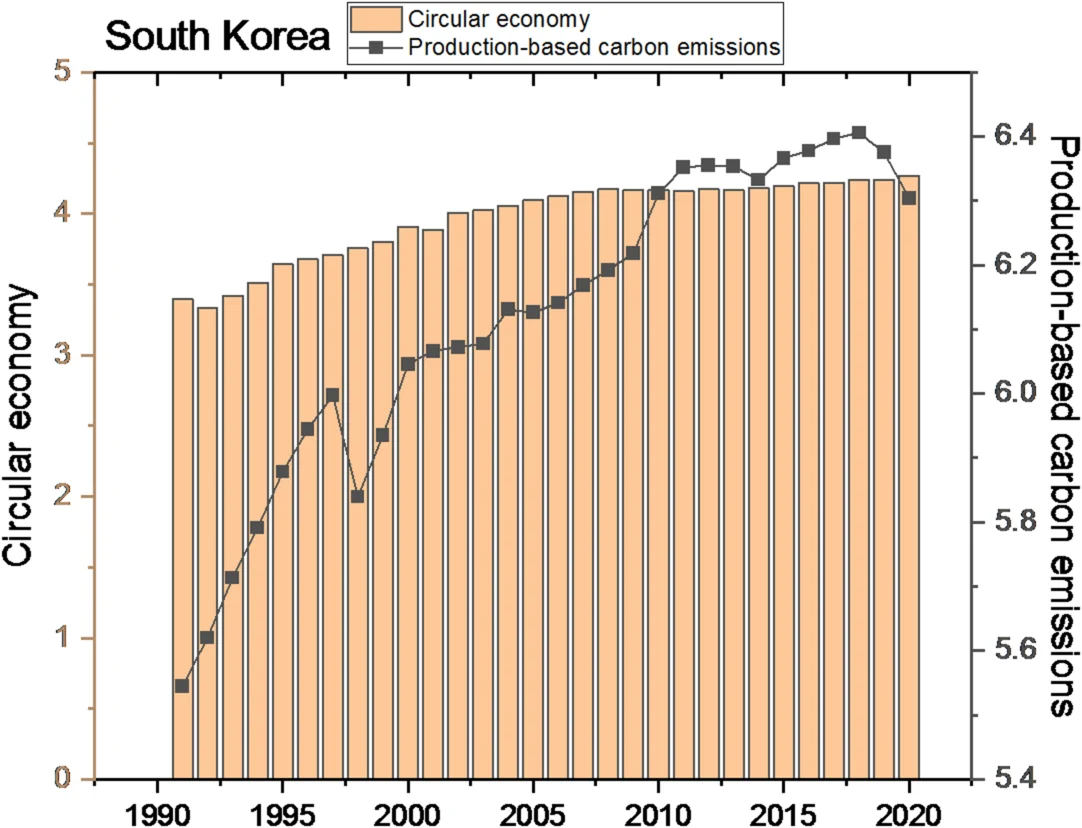
PBC and CE relationship visualization of South Korea from 1991 to 2020.

**Fig. 11:**
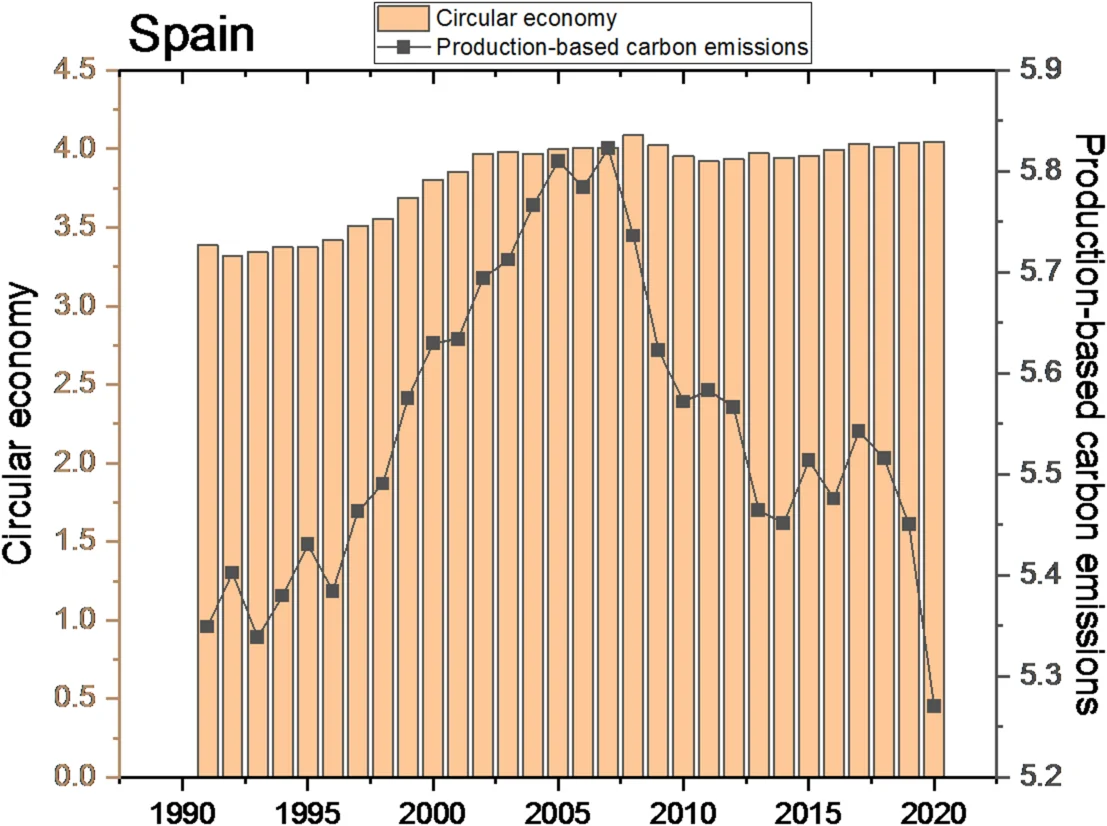
Visualizing the correlation between PBC and CE of Spain from 1991 to 2020.

**Fig. 12:**
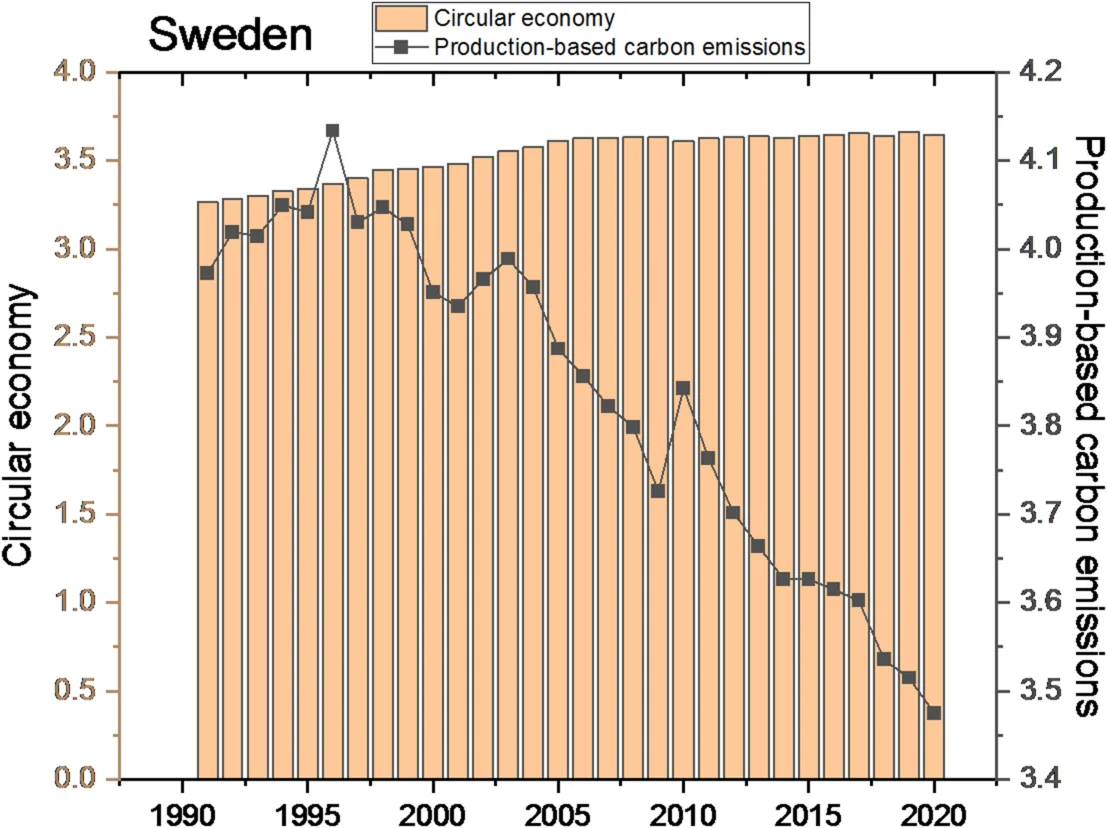
A visual representation of PBC-CE dynamics of Sweden from 1991–2020.

**Fig. 13:**
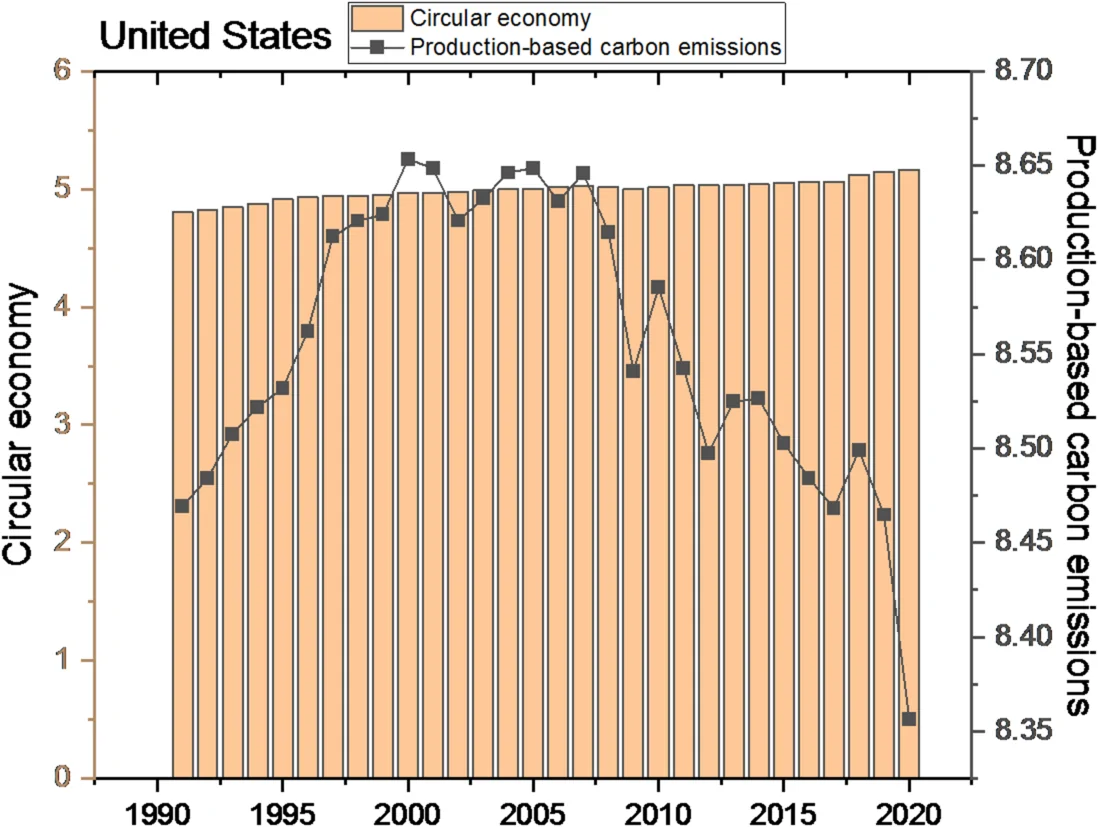
Illustrating the relationship between PBC and CE of United States from 1991 to 2020.

Table 6 showcases the long-term effects of various explanatory variables on PBC in each of the circular economy individually. Figures 8, 9, 10, 11, 12 and 13 deliver insightful visual representation of the dynamic relationship between CE and PBC for each country under analysis. This graphical display is crucial in revealing the ongoing trends and interplay between both factors, contributing to a deeper understanding of their complex interaction within various national contexts. The analysis demonstrated a significant and negative correlation between circular economy and PBC across most of the examined nations, emphasizing circular economy critical role in promoting the environmental well-being. The rationale underlying the positive and negative signs of the coefficients is as follows;

Claudio-Quiroga and Poza,^78^ stated that Belgium, Sweden, and the USA are in the forefront in establishing strategies with CE processes. These countries have enhanced the application of secondary raw resources and waste management practices. In the meantime, Austria and USA are formulating and executing strategies to speed up the move to a circular economy and attain carbon neutrality^79^. In comparison to the chosen countries, Spain behind the top-performing nations. Spain aligns with several EU nations but lags in recycling efficiency due to lack of recycling infrastructure^80^. The structural challenge is the substantial amount of mixed garbage, which hinders obtaining of pure recyclable streams and rises operating expenses (Ecostar,^81^). To address this issue, Spain needs strengthen source separation and public awareness initiatives.

The correlation between R&D and emissions resulting from production in certain advanced nations can be complicated. It is possible that investments in research and development will result in the creation of new industries or the expansion of existing ones, which will produce higher levels of production and possibly higher levels of emissions.

Moreover, the negative coefficients for natural resources rent indicate that natural resource rents have the potential to reduce carbon emissions through a variety of channels, including investments in renewable energy, the promotion of sustainable practices, and the utilization of carbon capture and storage technologies. Divergent tendencies can be observed in the relationship between the Human Development Index and the influence of information and communication technology on production-based emissions within the context of circular economies. It is important to note that the expansion of information and communications technology frequently results in an increase in production-based emissions, whereas improvements in human development index are typically associated with a decrease in such emissions. These findings shed light on the complex nature of the relationship that exists between socio-economic factors, technological advancement, and environmental outcomes in economies that place an emphasis on the utilization of resources in a circular and sustainable manner.

**Table 6:** Country-wise long run results by FMOLS for 06 circular economies from 1991 to 2020.

	Austria	Belgium	South Korea	Spain	Sweden	USA
Variables	Coeff.	Coeff.	Coeff.	Coeff.	Coeff.	Coeff.
logCE	-0.0509**	-0.3201*	-0.0375*	0.0943	-0.9130*	-0.2547*
logRD	-0.2661*	0.2769*	0.3618*	0.2412*	0.0933	-0.1275**
logNR	0.0841*	-0.0796*	-0.0372*	-0.1402***	-0.0053	0.0425*
logICT	0.0653*	0.1496*	0.0491*	0.1059*	0.2288*	0.0881*
logHDI	1.2958	-9.6082*	-0.9902*	-7.0121*	-5.5422*	-2.0045*
R-Squared	0.6193	0.9105	0.9668	0.8058	0.9153	0.8740
Obs. after adjustment	29	29	29	27	29	29

Note: Asterisks *, ** and *** demonstrate significance level at 1%, 5% and 10% respectively.

### The outcomes of country-wise short-run-ARDL-ECM

This academic endeavor employs the ARDL_ECM short-run methodology to anticipate long-term parameters proactively. Table 7 effectively summarizes the short-run results obtained. The analysis encompasses a historical examination of 06 circular economies from 1991 to 2020. Notably, the study reveals and suggests a mixed interaction between circular economy and production-based emissions. The outcomes of this analysis provide promising indications that a circular economy holds significant potential in the short term for reducing production-based emissions. Additionally, the control variables, including R&D, natural resource rent, information communication technology and human development demonstrate influence on PBC across the panel in the long-run. In summary, ARDL_ECM short-run approach provides valuable insights into the relationships between circular economy, control variables, and production-based carbon emission, highlighting the potential benefits of circular economy in advancing sustainable solutions.

**Table 7:** ARDL-Based Error Correction Model for 06 circular economies from 1991 to 2020.

Variables	Austria	Belgium	South Korea	Spain	Sweden	USA
	Coeff.	Coeff.	Coeff.	Coeff.	Coeff.	Coeff.
logCE	0.166	-0.2076**	0.0726	-0.0004	0.1488	-0.0946
logRD	0.2758	0.7144*	0.0351	0.2932	-0.1892***	0.8185*
logNR	-0.0760***	-0.0431**	-0.0387**	0.0753***	0.0535***	0.0143
logICT	-0.1716*	0.0785*	0.0752**	-0.0478	0.2564**	-0.0054
logHDI	8.4036***	-7.4469*	-1.9679	5.6231***	-0.7864	4.6589*
ECM(t-1)	-0.5385*	-0.9708*	-0.3094*	-0.7374*	-0.6375**	-0.5337*
R-Squared	0.6201	0.837	0.9074	0.7112	0.7699	0.7567

Note: *, **, and *** illustrate significance levels at 1%, 5%, and 10% respectively.

### The result of the stability test for ARDL_ECM short-run

**Fig. 14:**
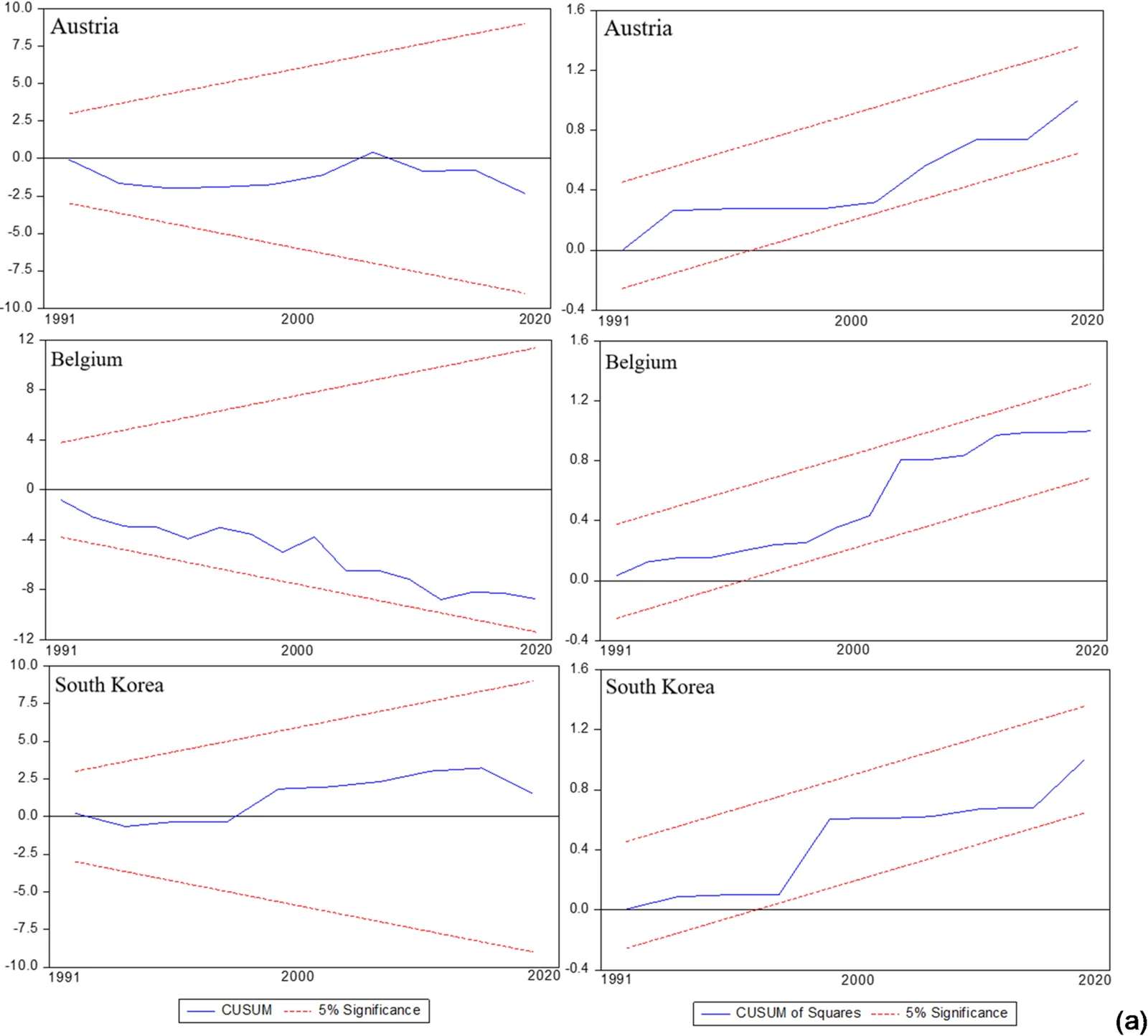

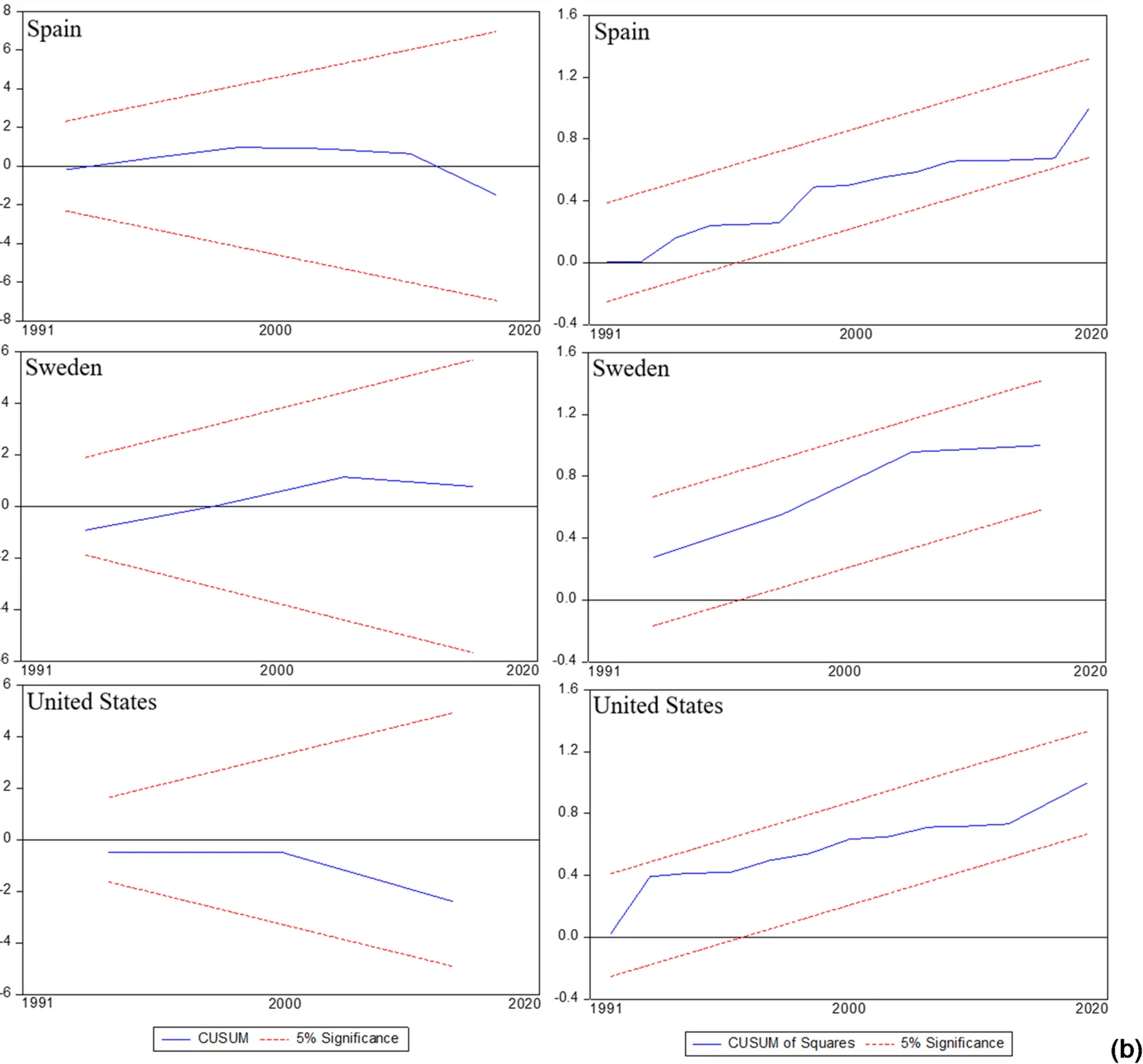
**(a).** illustrates the visualization of the CUSUM and CUSUM Squares stability tests of Austria, Belgium, and South Korea. **(b).** Illustrates the visualization of the CUSUM and CUSUM Squares stability tests of Spain, Sweden and United States.

Table 8 presents robustness analysis through stability examinations, revealing that the coefficient estimates derived from the Lagrange Multiplier (LM), Autoregressive Conditional Heteroskedasticity (ARCH), and Breusch-Pagan (BP) tests collectively indicate an absence of serial correlation and heteroscedasticity within the study. Furthermore, employing the listed stability test provides substantial evidence of the reliability of estimation, thereby enhancing the credibility and trustworthiness of the drawn conclusions. It is imperative to mention that “S” reports that the tests are stable. Figure 14 (a) and 14 (b) present the pictorial work of CUSUM and CUSUM Squares stability tests of the chosen countries.

**Table 8:** Stability Checks for ARDL_ECM (short-run).

	Austria	Belgium	S. Korea	Spain	Sweden	USA
Normality	0.7625	0.2363	0.6005	0.3229	0.7472	0.7343
Durbin-Watson	2.4476	2.1462	1.879	2.1234	2.0179	2.3425
LM Serial Correlation	0.5188	0.8369	0.4685	0.7055	0.5464	0.3037
ARCH	0.4675	0.7692	0.4086	0.1965	0.9746	0.6843
BPG	0.3398	0.9552	0.4889	0.7738	0.906	0.3059
CUSUM & CUSUM square	S	S	S	S	S	S
Forecast	S	S	S	S	S	S
Recursive	S	S	S	S	S	S

### The result of long-run pairwise D-H panel causation

Table 9 presents the findings of a Dumitrescu-Hurlin (D-H) long-run Granger causality analysis from 1991 to 2020, focusing on six circular economies. This analysis examines the causal relationships between various factors within these economies over the specified time, offering valuable insights into their dynamics and potential impacts on sustainable development. This study reveals a unidirectional causal relationship between the circular economy, R&D, ICT, HDI, and production-based carbon emissions in circular economies. These findings underscore the pivotal role played by the circular economy, R&D, ICT, and HDI in influencing carbon emissions resulting from production processes within economies that prioritize sustainable resource use.

Unidirectional causality demonstrates that CE practices considerably determine emission levels. The underlying causes of this unidirectional relationship are predominantly attributable to the way resource management significantly alters production dynamics. Circular solutions (recycling, reuse, and remanufacturing) immediately reduce the necessity for raw material extraction and energy-intensive processing, which are principal drivers of PBC. Extending the lifespan of materials and goods allows the economy to preserve the “embodied energy” invested in their initial manufacturing.

The unidirectional causality observed in this research suggests that advancements in circular economy practices, R&D investments, ICT development, and improvements in HDI can effectively drive down production-based carbon emissions. This highlights the importance of fostering innovation, human development, and a transition towards circular economic models to mitigate the environmental impact of industrial activities. Furthermore, these findings emphasize the significance of considering the interplay between various socio-economic factors and technological advancements when designing policies and strategies aimed at achieving sustainable development and reducing carbon emissions.

**Table 9:** Pairwise causality.

Causal directions	W-Stat.	Zbar-Stat.	Prob.
logCE to logPBC	4.4768**	2.3237	0.0201
logPBC to logCE	2.9974	0.8201	0.4121
logRD to logPBC	17.6032*	4.0588	0.0001
logPBC to logRD	10.6626	1.3158	0.1882
logNR to logPBC	5.1573*	3.0154	0.0026
logPBC to logNR	4.2206**	2.0633	0.0391
logICT to logPBC	6.7130*	2.6550	0.0079
logPBC to logICT	5.0322	1.3346	0.1820
logHDI to logPBC	5.6750*	3.5416	0.0004
logPBC to logHDI	2.3241	0.1358	0.8920
logRD to logCE	3.8010	1.6369	0.1017
logCE to logRD	7.9429*	5.8465	0.0000
logNR to logCE	2.7537	0.5725	0.5670
logCE to logNR	3.4307	1.2605	0.2075
logICT to logCE	4.4517**	2.2982	0.0216
logCE to logICT	6.4469*	4.3260	0.0000
logHDI to logCE	3.4802	1.3109	0.1899
logCE to logHDI	1.9462	-0.2483	0.8039
logNR to logRD	2.6192	0.4358	0.6630
logRD to logNR	4.2794**	2.1232	0.0337
logICT to logRD	8.3491*	6.2594	0.0000
logRD to logICT	8.6545*	6.5698	0.0000
logHDI to logRD	8.4013*	6.3124	0.0000
logRD to logHDI	2.1828	-0.0078	0.9937
logICT to logNR	1.5900	-0.6103	0.5416
logNR to logICT	1.7263	-0.4718	0.6371
logHDI to logNR	2.8910	0.7120	0.4765
logNR to logHDI	1.6102	-0.5898	0.5553
logHDI to logICT	2.6331	0.4498	0.6528
logICT to logHDI	1.0326	-1.1769	0.2392

Note: *, **, and *** refer to levels of significance at 1%, 5%, and 10% respectively.

## Conclusion

This study’s primary objective is to investigate the effects of circular economy on PBC in circular economies, taking into account the research and development, natural resource rent, information communication and technology, and human development. The analysis covers the period from 1991 to 2020 and employs the ARDL-PMG, ARDL-MG, and ARDL-DFE estimation method to examine long-run relationships among the variables. In the context of six advanced circular economies, the circular economy model contributes to reducing production-based carbon emissions. Additionally, investments in research and development, management of natural resource rents, and improvements in human development also demonstrate a positive impact on mitigating production-based carbon emissions. However, ICT has been observed to increase production-based emissions within this panel of advanced circular economies in the long run. These findings highlight the importance of transitioning towards a circular economy, fostering innovation, managing resources sustainably, and advancing human development to effectively address environmental challenges. At the same time, they underscore the need for policy interventions to ensure that the growth and implementation of ICT align with broader sustainability goals and minimize negative impacts on carbon emissions.

### Policy implications

Through the implementation of these policies, governments can establish a favorable environment for the shift towards a circular economy. This will allow them to effectively tackle emissions that result from production activities, while also promoting sustainable development and the well-being of society.

First, urge the implementation of extended producer responsibility regulations to obligate producers to take responsibility for the entire life cycle of their products, including recycling and disposal. This will serve as an incentive for the development of supplementary environmentally responsible products and reduce the emissions that are generated during the production process.

Second, the study pushes for the prohibition or substantial taxation of single-use plastics and non-recyclable packaging to diminish waste and promote the adoption of recyclable or biodegradable alternatives.

Third, establish specialist recycling facilities in countries equipped with advanced infrastructure and skills to process complex products, including electronics, automobile components, ships, and cargo, emphasizing the extraction of rare materials. This will enable the reutilization of these materials in renewable energy, carbon capture technologies, and other sectors to more efficiently tackle environmental concerns.

Third, provide financial incentives, grants, and tax advantages to promote investments in the research and development of circular economy technologies, materials, and practices that contribute to the reduction of carbon emissions in manufacturing processes.

Fourth, develop and implement educational programs, vocational training, and public awareness campaigns to provide residents with the essential knowledge and skills for a successful transition to a circular economy. This will foster the generation of innovative concepts and the use of sustainable industrial techniques.

Fifth, promote the adoption of digital technologies, such as Internet of Things (IoT), big data, and artificial intelligence (AI), to maximize resource utilization, enhance waste management, and optimize overall efficiency in production processes. Monitor and address any potential adverse environmental effects linked to the expansion of information and communication technology (ICT).

Sixth, enact policies that encourage the effective utilization of natural resources, which includes sustainable extraction methods and equitable allocation of resource revenues. Implementing this approach will guarantee a harmonious equilibrium between economic progress and safeguarding the environment, ultimately leading to a decrease in emissions resulting from production activities.

Finally, within the context of Spain, the “Anticipa” project can function within the framework of Spain, representing a coordinated effort to achieve recycling objectives in the country. The initiative aims to foresee and tackle difficulties associated with infrastructure, legislation, and recycling practices across the region. “Anticipa” seeks to create a sustainable and efficient waste management framework through a comprehensive approach encompassing public awareness and technology upgrading.

### Study limitation and future direction

Although this study provides valuable insights into the correlation between circular economy, production-based emissions, and different control variables, it does have some limitations. The analysis utilizes data from a specific temporal range (1991–2020) and concentrates on only six advanced circular economies, potentially constraining the applicability of the results. Moreover, the study predominantly utilizes quantitative data and the ARDL-PMG methodology, the outcomes of the research may be subject to variation if different methodologies, different selection of sample countries, study timeframe, and estimation techniques are employed. The findings of this study provide numerous opportunities for future research. Additional research could explore the causal connections between circular economy practices and emissions resulting from production. It could also examine the potential influence of research and development, human development, ICT, and natural resource rent as mediating factors. Furthermore, future research could investigate the suitability of the ARDL-PMG methodology in various contexts and economic sectors, thereby enhancing our understanding of the interplay between these variables. Both long-run estimate methods mitigate endogeneity, while quantile regression may be utilized for further analyses.

## Data Availability

Data will be provided upon reasonable request from the corresponding authors.
